# Abnormal intrinsic functional hubs and connectivity in patients with post‐stroke depression

**DOI:** 10.1002/acn3.52091

**Published:** 2024-05-22

**Authors:** Xiumei Wu, Kang Xu, Tongyue Li, Luoyu Wang, Yanhui Fu, Zhenqiang Ma, Xiaoyan Wu, Yiying Wang, Fenyang Chen, Jinyi Song, Yulin Song, Yating Lv

**Affiliations:** ^1^ Center for Cognition and Brain Disorders The Affiliated Hospital of Hangzhou Normal University Hangzhou Zhejiang China; ^2^ Zhejiang Key Laboratory for Research in Assessment of Cognitive Impairments Hangzhou Zhejiang China; ^3^ School of Biomedical Engineering ShanghaiTech University Shanghai China; ^4^ Department of Neurology Anshan Changda Hospital Anshan Liaoning China; ^5^ Department of Image Anshan Changda Hospital Anshan Liaoning China; ^6^ Department of Ultrasonics Anshan Changda Hospital Anshan Liaoning China; ^7^ The Fourth Clinical Medical College Zhejiang Chinese Medical University Hangzhou Zhejiang China; ^8^ III Department of Clinic Medicine Zhejiang University Hangzhou Zhejiang China

## Abstract

**Objective:**

The present study aimed to investigate the specific alterations of brain networks in patients with post‐stroke depression (PSD), and further assist in elucidating the brain mechanisms underlying the PSD which would provide supporting evidence for early diagnosis and interventions for the disease.

**Methods:**

Resting‐state functional magnetic resonace imaging data were acquired from 82 nondepressed stroke patients (Stroke), 39 PSD patients, and 74 healthy controls (HC). Voxel‐wise degree centrality (DC) conjoined with seed‐based functional connectivity (FC) analyses were performed to investigate the PSD‐related connectivity alterations. The relationship between these alterations and depression severity was further examined in PSD patients.

**Results:**

Relative to both Stroke and HC groups, (1) PSD showed increased centrality in regions within the default mode network (DMN), including contralesional angular gyrus (ANG), posterior cingulate cortex (PCC), and hippocampus (HIP). DC values in contralesional ANG positively correlated with the Patient Health Questionnaire‐9 (PHQ‐9) scores in PSD group. (2) PSD exhibited increased connectivity between these three seeds showing altered DC and regions within the DMN: bilateral medial prefrontal cortex and middle temporal gyrus and ipsilesional superior parietal gyrus, and regions outside the DMN: bilateral calcarine, ipsilesional inferior occipital gyrus and contralesional lingual gyrus, while decreased connectivity between contralesional ANG and contralesional supramarginal gyrus. Moreover, these FC alterations could predict PHQ‐9 scores in PSD group.

**Interpretation:**

These findings highlight that PSD was related with increased functional connectivity strength in some areas within the DMN, which might be attribute to the specific alterations of connectivity between within DMN and outside DMN regions in PSD.

## Introduction

As one of the most prevalent psychological complications after stroke, post‐stroke depression (PSD) affects around one‐third of stroke survivors.^1^, ^2^ Patients suffering from PSD are often related to delayed functional recovery and increased risk of stroke recurrence and mortality.^3^, ^4^, ^5^, ^6^ Therefore, understanding the brain mechanisms underlying the PSD would provide supporting evidence for early diagnosis and interventions for the disease.

The developments of resting‐state‐functional magnetic resonance imaging (rs‐fMRI) have allowed spontaneous neural activity to be well characterized.^7^ To date, rs‐fMRI has been employed to detect the local functional abnormalities in PSD.^8^, ^9^, ^10^, ^11^ For example, our previous study found that PSD‐specific alterations of local neural activity were frequency‐dependent and time‐variant.^11^ However, these studies mainly focused on elucidating the neural activity changes in specific brain areas. Accumulating evidence has indicated that the human brain is organized essentially as a network that integrates information processing among distributed components.^12^ Thus, the connectivity‐based analytic approaches are more appropriate to explore the alterations of the functional brain networks, especially for investigating how the brain responds to focal damages after stroke.

Seed‐based functional connectivity (FC), calculated by the temporal correlation between spatially distinct brain regions, has been widely applied to examine the associations between brain regions within neural networks/circuits.^13^, ^14^ Using this approach, previous studies found that the altered connectivity in default mode network (DMN), affective network, and cognitive control network might be relevant to PSD.^15^, ^16^, ^17^, ^18^ However, the seeds predefined for FC analyses in these studies were based on the disruptions in major depression disorder. It is thus necessary to use the seeds that exhibited PSD‐specific connectivity changes to figure out the alterations of neural networks in PSD. Recently, Fan and colleagues in our group adopted the dorsolateral prefrontal cortex as the seed, the center of the depression circuit that lesion locations of PSD mapped to,^19^ and found PSD underwent specific alterations in the depression circuit.^20^ Therefore, further questions would be whether PSD‐specific alterations exist in other neural networks/circuits and how to select the seeds to examine the connectivity changes in these networks. Degree centrality (DC), as a voxel‐wise data‐driven method, offered a viable solution for the latter question. With treating each voxel as a node and the functional connection between each pair of voxels as an edge, DC calculates the number or strength of direct connections for a given node and reflects the connectivity importance of this node in the whole‐brain network.^21^ Without priori selection of nodes or seeds, this approach identifies “hubs” with a high degree centrality at the voxel level, and the “hubs” are considered to be crucial in efficient communication.^12^ DC has been utilized as a marker for altered functional connectivity in neuropsychiatric diseases.^22^, ^23^, ^24^ Thereby, a combination of voxel‐wise DC and seed‐based FC approaches may be an effective strategy for exploring the specific alterations of brain networks in PSD.

In this study, by employing resting‐state fMRI data from nondepressed stroke patients, PSD patients, and healthy controls, we aimed to investigate the specific alterations of brain networks in PSD. We first performed voxel‐wise DC to detect regions showing PSD‐specific changes in network centrality. Subsequently, FC analyses with the seeds selected based on these regions were conducted to investigate the PSD‐related connectivity changes in different brain networks. Finally, the relationship between these alterations and depression severity was further examined in PSD patients.

## Materials and Methods

### Participants

A total of 127 patients with first‐time ischemic stroke were recruited from the Department of Neurology, Anshan Changda Hospital. Patients included 88 nondepressed stroke patients (Stroke), and 39 post‐stroke depression patients (PSD) diagnosed by professional neurologists according to the Diagnostic Statistical Manual of Mental Disorder, Fifth Edition (DSM‐5) criteria. That is, PSD patients with stroke‐caused depression disorder must have depressed mood or loss of interest or pleasure along with at least two but less than five symptoms of major depression.^25^ Each patient did not take any antidepressants and met the following inclusion criteria: (1) diagnosis of ischemic stroke by neurologists; (2) admitted <1 month after stroke onset; (3) unilateral focal brain lesions; (4) confirmed no depression before stroke onset by asking the medical history. Patients with other psychiatric disease history, hemorrhage, epilepsy, or migraine were excluded.

In addition, 74 age‐ and gender‐matched healthy controls (HC) were recruited from the local community. None of the HCs had a history of physical or psychiatric diseases.

Data from six nondepressed stroke patients were excluded due to excessive head motion (see preprocessing of rs‐fMRI data). Leaving 82 patients in the Stroke group (32 females, 59.41 ± 6.45 years), 39 patients in PSD group (18 females, 60.00 ± 9.41 years), and 74 healthy controls in the HC group (31 females, 57.43 ± 6.83 years) in the final analyses.

The study was approved by the Ethics Committee of the Center for Cognition and Brain Disorders, Hangzhou Normal University, and all participants signed informed consent.

### Data acquisition

#### Clinical data collection

Within 24 hours before the MRI scan, participants received the following assessments: National Institutes of Health Stroke Scale (NIHSS) that reflects the stroke severity of patients;^26^, ^27^ Activity Daily Living Scale (ADL) that assesses the patients' self‐care ability and mobility;^26^ Patient Health Questionnaire‐9 (PHQ‐9),^28^ Hamilton Depression Rating Scale (HAMD),^29^ and Center for Epidemiological Survey Depression Scale (CES‐D)^30^ that reflect the depression severity in participants.

#### MRI data acquisition

Rs‐fMRI, structural MRI (sMRI), and diffusion‐weighted imaging (DWI) data were collected from all participants using a 3 T scanner (GE MR‐750, Waukesha, WI) at Anshan Changda Hospital (see Supplementary Materials for parameters of MRI data acquisition).

### Data preprocessing

#### Processing of sMRI image and lesion map

**Figure 1:**
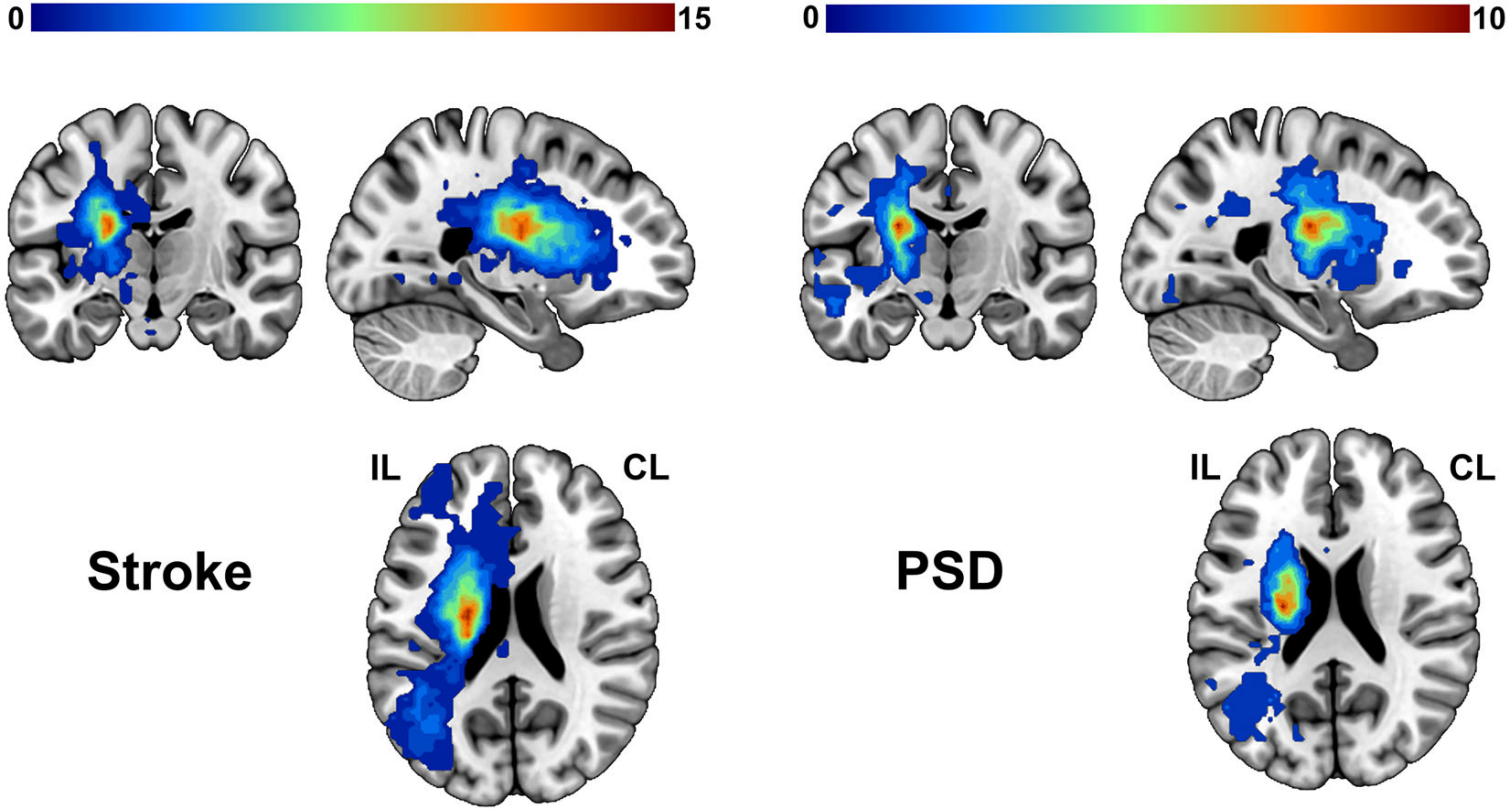
Overlap maps of lesions. Lesions were overlapped in Stroke (left) and PSD groups (right). The color depicts the number of patients with focal brain lesion at any given location. CL, contralesional hemisphere; IL, ipsilesional hemisphere; PSD, poststroke depression; Stroke, nondepressed stroke patients.

According to the high‐resolution sMRI and DWI images for each patient, one experienced radiologist (F.C.) manually traced lesion masks using ITK‐SNAP software (https://www.itksnap.org). The lesion masks were smoothed with 3 mm full‐width half maximum (FWHM) to remove jagged edges.^31^ Subsequently, the lesion masks, DWI, and sMRI images from patients with a right‐hemispheric lesion were flipped to left hemisphere. Due to distortion of lesion for the segmentation and normalization of the sMRI image, the abnormal values within the lesion were replaced by values from the contralesional homologous regions using clinicaltbx (https://www.nitrc.org/plugins/mwiki/index.php/clinicaltbx:MainPage) based on Statistical Parametric Mapping (SPM).^32^ The corrected sMRI images were then segmented and normalized to the Montreal Neurological Institute (MNI) space to obtain deformation information. Two lesion masks from sMRI and DWI images were further normalized to the MNI space via deformation fields derived from tissue segmentation of sMRI images for each patient. Finally, the union of two masks was calculated to obtain the lesion map for each patient. Overlapping lesions for Stroke patients and PSD patients are displayed in Figure 1.

#### Preprocessing of rs‐fMRI data

The rs‐fMRI data were preprocessed using SPM12 (http://www.fil.ion.ucl.ac.uk/spm) and Data Processing and Analysis for Brain Imaging (DPABI, http://rfmri.org/dpabi) based on the MATLAB platform. The preprocessing steps included: (1) The fMRI data of patients with right‐hemispheric lesions were flipped to the left hemisphere, making the left hemisphere correspond to the ipsilesional hemisphere;^33^, ^34^, ^35^ (2) removing the initial 10 volumes; (3) slice timing; (4) correcting intervolume head motion. Participants with maximal displacement >3 mm, rotation >3°, or mean framewise displacement (FD) >0.5^36^ were excluded; (5) spatial normalization to the MNI space via deformation fields derived from tissue segmentation of sMRI images as described above; (6) spatial smoothing by 6‐mm FWHM isotropic Gaussian kernel. Notably, smoothing was not performed when calculating the DC; (7) removing linear trends; (8) making individual white matter (WM) and cerebrospinal fluid (CSF) masks: 95% probability masks of individual WM and CSF obtained from segmentation of sMRI image were first normalized to MNI space. The normalized masks were first intersected with WM and CSF template in DPABI and then removed the lesion for each patient to obtain individual WM mask and CSF mask; (9) regressing out 24 head motion parameters,^37^ WM and CSF signals. The global signals did not regress out for consideration of its potential impact on rs‐fMRI data.^38^ (10) making gray matter mask for all participants: the smoothed fMRI data were used to extract the whole brain mask by the function “w_Automask” in DPABI. The intersection of all brain masks was further intersected with the anatomical automatic labeling (AAL) atlas with eight subcortical regions and lesions removed to obtain the gray matter mask; (11) correcting hemodynamic lags of each stroke patient by time‐shift analysis,^39^ in consideration of the effect of hemodynamic lags on the functional connectivity in ischemic stroke patients;^40^, ^41^, ^42^, ^43^ and (12) band‐pass filtering (0.01–0.08 Hz).

### Degree centrality

Voxel‐wise DC analysis was performed in RESTplus software (http://restfmri.net/forum/RESTplus).^44^ DC map was obtained by calculating the Pearson's correlation coefficient of time series between each voxel with all other voxels and counting the number of connections above the threshold of *r* > 0.32 (*p* < 0.05, Bonferroni corrected over whole‐brain voxels). Subsequently, the DC map was normalized by dividing the mean DC of the map and smoothed with a 6‐mm FWHM Gaussian kernel for further analysis.

### Seed‐based functional connectivity

Seed‐based FC analysis was conducted using DPABI (http://rfmri.org/dpabi).^45^ Seeds were defined as the regions that exhibited PSD‐specific DC alterations. For each seed, the averaged time course was first extracted from the preprocessed rs‐fMRI data. FC map was then acquired by computing the Pearson's correlation coefficients between the averaged time course of each seed region and time courses of all other voxels in the brain. The FC map was further normalized with Fisher's r‐to‐z transformation for the following analyses.

### Statistical analysis

#### Group difference in demographic and clinical characteristics analysis

All statistical analyses for demographic and clinical data were carried out in SPSS version 26.0 (http://www.spss.com/software/statistics). For continuous variables, after detecting the normality of data by the Shapiro–Wilk test, one‐way ANOVA or Kruskal–Wallis test was used for comparison among three groups (i.e., age), Mann–Whitney *U*‐test was used to assess differences between two groups (i.e., NIHSS scores, lesion size). For categorical variables, chi‐square tests were used for evaluating intergroup differences (i.e., gender).

#### Group difference in degree centrality

Using DPABI (http://rfmri.org/dpabi),^45^ one‐way ANOVA analyses were applied to compare the DC differences among Stroke, PSD, and HC groups with gender and age as covariates of noninterest in the gray matter mask obtained. The resultant F map was corrected by a voxel‐level false discovery rate (FDR) procedure for multiple comparisons,^46^, ^47^ with a threshold of *q* < 0.05 and a spatial contiguity criterion of 20 adjacent voxels (k > 20 voxels).

The simple effect analyses (two‐sample *t*‐test) were conducted to further identify differences in DC between each pair of groups within the mask obtained from the significant results of one‐way ANOVA analyses. Age and gender were used as covariates in the comparisons between Stroke and HC groups and between PSD and HC groups, while NIHSS scores, age, and gender were treated as covariates in the comparison between PSD and Stroke groups (*q* < 0.05, FDR corrected, k > 20 voxels).

For each region with significant differences between PSD and Stroke groups, the average DC values were compared between each pair of three groups in SPSS to identify the region of PSD‐specific DC alterations, that is, PSD group show higher or lower DC values than both Stroke and HC groups (*p* < 0.05, Bonferroni corrected).

#### Group difference in functional connectivity

Similarly, one‐way ANOVA analyses were applied to compare the FC differences among Stroke, PSD, and HC groups in which gender and age were regarded as covariates. The simple effect analyses were further inferred using two‐sample *t*‐test between each pair of groups (Stroke vs. HC, PSD vs. HC, and PSD vs. Stroke). Age and gender were also treated as covariates in the comparisons between Stroke and HC groups and between PSD and HC groups, whereas NIHSS scores, age, and gender were used as covariates of no interest in the comparison between PSD and Stroke groups (*q* < 0.05, FDR corrected, k > 20 voxels). After the group comparisons, the regions of PSD‐specific FC alterations were determined.

#### Correlation analysis

For any metric (DC and FC) showing PSD‐specific alterations, a Spearman correlation analysis was conducted to evaluate its associations with depression scale score (PHQ‐9, HAMD, and CES‐D) in PSD group, and significance was set at *p* < 0.05/3 (*p* < 0.017, Bonferroni corrected).

#### Prediction analysis

To explore whether the connectivity alterations could predict the depression severity in PSD, the ridge regression analyses were applied using FC values in the regions which showing PSD‐specific FC alterations based on the scikit‐learn library in Python (https://scikit‐learn.org/stable/). Specifically, the mean FC values of each region with PSD‐specific FC alterations in PSD group as the features. The optimal regularization coefficient lambda was determined by leave‐one‐out cross‐validation (LOOCV), resulting in a reliable ridge regression model for each depression scale score (PHQ‐9, HAMD, and CES‐D). Then the Spearman correlation coefficients were calculated between the real depression scale scores and the predicted depression scale scores to estimate prediction performance, with the significance level set at *p* < 0.05/3 (*p* < 0.017, Bonferroni corrected).

## Results

### Group difference in demographics and clinical characteristics

There were no significant differences in age and gender (*p* > 0.05) among three groups, while significant differences were found in PHQ‐9 scores and ADL scores (*p* < 0.001). PSD and Stroke groups did not differ significantly in terms of lesion hemisphere and lesion size (*p* > 0.05). Compared to Stroke group, PSD group exhibited significantly longer duration of the stroke and higher NIHSS, HAMD, and CES‐D scores (*p* < 0.001). Stroke group had PHQ‐9 scores of 0–4 (no depression), HAMD scores of 0–4 (no depression), and CES‐D scores of 11–15 (no depression). Whereas PSD group had PHQ scores of 5–18 (mild to moderately severe depression), HAMD scores of 17–23 (moderate depression), and CES‐D scores of 17–38 (depression). Demographic and clinical detailed information are presented in Table 1.

**Table 1:** Demographic and clinical characteristics of participants.

	Stroke	PSD	HC	*p* value
	(*n* = 82)	(*n* = 39)	(*n* = 74)	
Gender (M/F)	50/32	21/18	43/31	0.756^a^
Age (years)	59.41 ± 6.45	60.00 ± 9.41	57.43 ± 6.83	0.121^b^
Onset time (days)^e^	4.85 ± 3.26	9.92 ± 4.92		<0.001^c^
Lesion hemisphere (L/R)	44/38	23/16		0.582^a^
Lesion size (voxels)	128.42 ± 256.28	128.94 ± 178.17		0.206^c^
PHQ‐9	0.23 ± 0.69	8.23 ± 2.94	0.69 ± 1.18	<0.001^d^
ADL	82.80 ± 16.76	67.05 ± 18.05	100.00 ± 0.00	<0.001^d^
NIHSS	2.54 ± 2.18	5.18 ± 3.49		<0.001^c^
HAMD	0.23 ± 0.79	19.23 ± 2.01		<0.001^c^
CES‐D	12.12 ± 0.57	24.44 ± 4.45		<0.001^c^

PHQ‐9 post‐hoc *t*‐tests: PSD vs HC (*p* < 0.001); PSD vs Stroke (*p* < 0.001). ADL post‐hoc *t*‐tests: Stroke vs HC (*p* < 0.001); PSD vs HC (*p* < 0.001); PSD vs Stroke (*p* < 0.001).

ADL, Activity Daily Living Scale; CES‐D, Center for Epidemiological Survey Depression Scale; F, female; HAMD, Hamilton Depression Rating Scale; HC, healthy controls; L, left; M, male; NIHSS, National Institutes of Health Stroke Scale; PHQ‐9, Patient Health Questionnaire‐9; PSD, poststroke depression; R, right; Stroke, nondepressed stroke patients.

^a^ The *p*‐value was obtained by a chi‐square test.

^b^ The *p*‐values were obtained by one‐way analysis of variance tests.

^c^ The *p*‐values were obtained by Mann–Whitney *U*‐test.

^d^ The *p*‐values were obtained by Kruskal–Wallis test.

^e^ Onset time (days) refers to the time between stroke onset and scanning.

### Group differences in degree centrality

**Figure 2:**
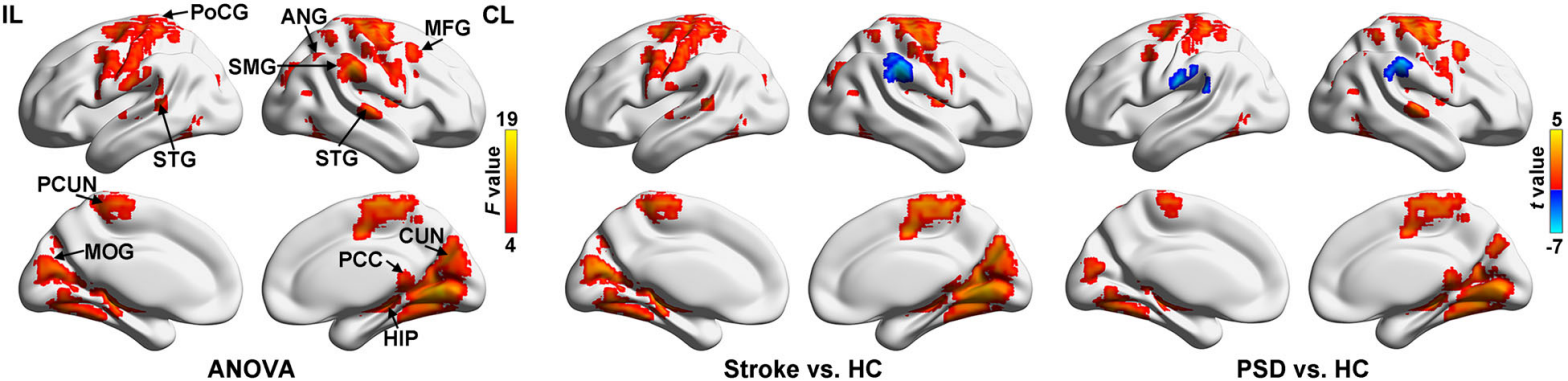
Group differences of DC. Brian regions showing significant differences in DC among the three groups (left), between Stroke and HC groups (middle) and between PSD and HC groups (right) (*q* < 0.05, FDR corrected, k > 20 voxels). ANG, angular gyrus; CL, contralesional hemishpere; CUN, cuneus; DC, degree centrality; HC, healthy controls; HIP, hippocampus; IL, ipsilesional hemishpere; MFG, middle frontal gyrus; MOG, middle occipital gyrus; PCC, posterior cingulate cortex; PCUN, precuneus; PoCG, postcentral gyrus; PSD, poststroke depression; SMG, supramarginal gyrus; STG, superior temporal gyrus; Stroke, nondepressed stroke patients.

One‐way ANOVA analyses revealed significant among‐group differences of DC in bilateral superior temporal gyrus (STG), ipsilesional middle occipital gyrus (MOG), postcentral gyrus (PoCG) and precuneus (PCUN), contralesional middle frontal gyrus (MFG), supramarginal gyrus (SMG), angular gyrus (ANG), posterior cingulate cortex (PCC), hippocampus (HIP), and cuneus (CUN) (*q* < 0.05, FDR corrected, k > 20 voxels) (Table S1 and Fig. 2).

**Figure 3:**
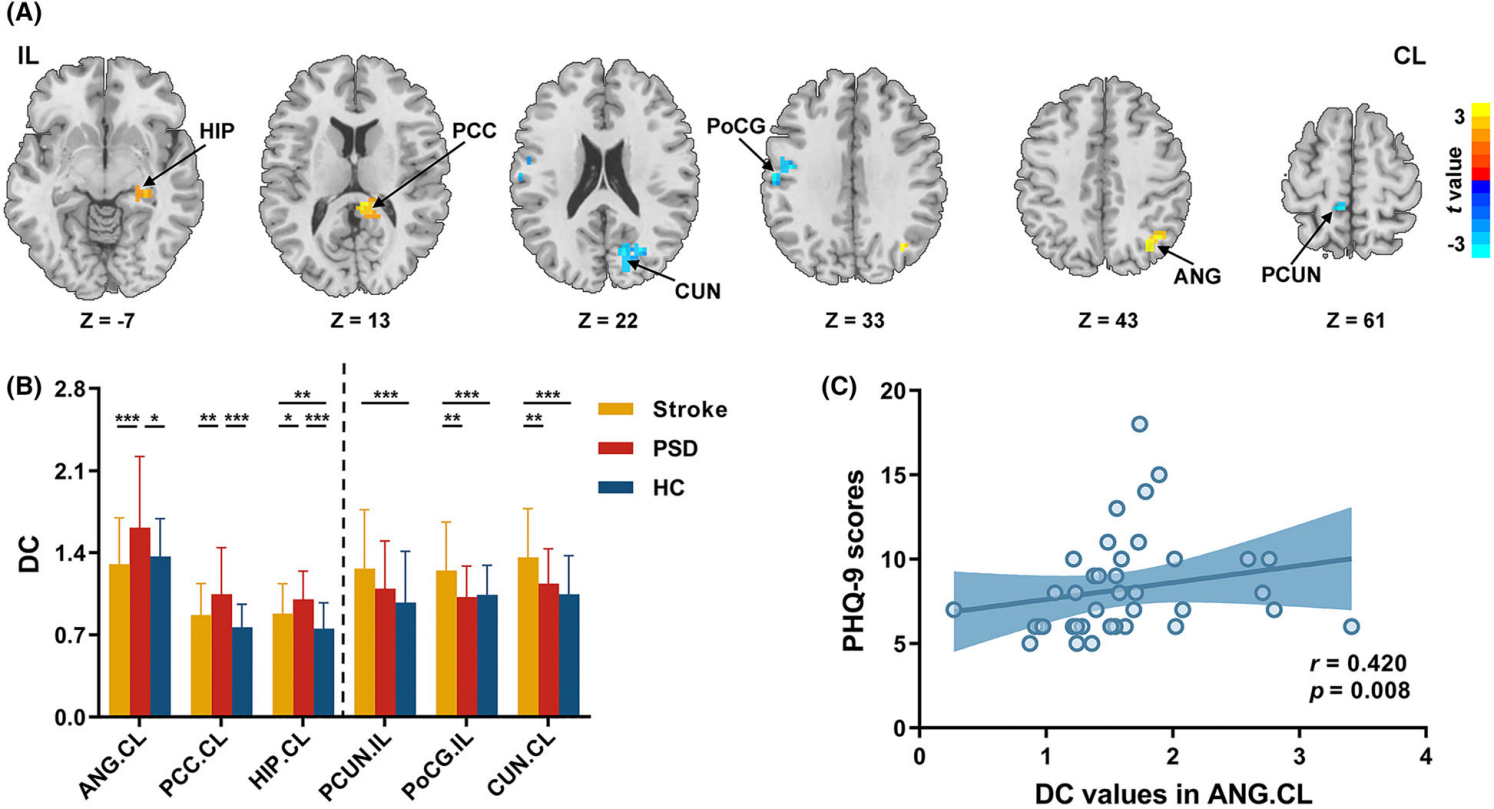
PSD‐specific region identified using DC. (A) The regions showing significant differences in DC between PSD and Stroke groups (*p* < 0.05, uncorrected, k > 20 voxels). Warm color indicates significantly higher DC value in PSD group than Stroke group. Cool color indicates significantly lower DC value in PSD group than Stroke group. (B) The bar graph shows comparisons of average DC values between each pair of three groups in brain regions with significant differences between PSD and Stroke groups (*p* < 0.05, Bonferroni corrected). The regions on the left of dashed line showing PSD‐specific DC alterations. (C) Significant correlation was observed between PHQ‐9 scores and contralesional ANG (*p* < 0.017, Bonferroni corrected). *p < 0.05; **p < 0.01; ***p < 0.001. ANG, angular gyrus; CL, contralesional hemishpere; CUN, cuneus; DC, degree centrality; HC, healthy controls; HIP, hippocampus; IL, ipsilesional hemishpere; PCC, posterior cingulate cortex; PCUN, precuneus; PHQ‐9, Patient Health Questionnaire‐9; PoCG, postcentral gyrus; PSD, post‐stroke depression; Stroke, nondepressed stroke patients.

Simple effect analyses revealed that the significant among‐group differences in DC were mainly attributed to the differences between Stroke and HC groups. That is, compared to HC group, both Stroke and PSD groups exhibited increased DC in ipsilesional MOG and PoCG, and contralesional MFG, HIP, PCC, and CUN, while decreased DC in contralesional SMG (*q* < 0.05, FDR corrected, k > 20 voxels) (Fig. 2). Compared with Stroke group, PSD group showed increased DC in contralesional ANG, PCC, and HIP and decreased DC in ipsilesional PoCG and PCUN, and contralesional CUN (*p* < 0.05, uncorrected, k > 20 voxels) (Table 2 and Fig. 3A). After further comparisons of DC values between each pair of three groups in these regions, PSD‐specific DC alterations were observed in contralesional ANG, PCC, and HIP in which PSD group showed higher DC relative to both Stroke and HC groups (*p* < 0.05, Bonferroni corrected) (Fig. 3B). Notably, no significant correlations with onset time were observed for DC values in regions showing PSD‐specific DC alterations (*p* > 0.05) (Table S2).

**Table 2:** Brain regions showing significant differences in DC between PSD and Stroke groups.

Cluster	MNI coordinate (mm)	Cluster size	*t* value	Brain region
1	30 −33 3	36	2.9114	HIP.CL
2	12 −36 9	43	3.0699	PCC.CL
3	−57 −18 36	66	−3.1207	PoCG.IL
4	15 −69 21	87	−2.7977	CUN.CL
5	36 −66 45	46	3.484	ANG.CL
6	−12 −36 72	26	−2.9619	PCUN.IL

ANG, angular gyrus; CL, contralesional hemisphere; CUN, cuneus; DC, degree centrality; HIP, hippocampus; IL, ipsilesional hemisphere; MNI, Montreal Neurological Institute; PCC, posterior cingulate cortex; PCUN, precuneus; PoCG, postcentral gyrus; PSD, poststroke depression; Stroke, nondepressed stroke patients.

### Group differences in functional connectivity

Based on the group comparison results, the three regions showing PSD‐specific DC alterations, that is, contralesional ANG, PCC, and HIP, were defined as the seed regions for the FC analyses (Table 2 and Fig. 5).

#### Contralesional ANG‐seeded FC

**Figure 4:**
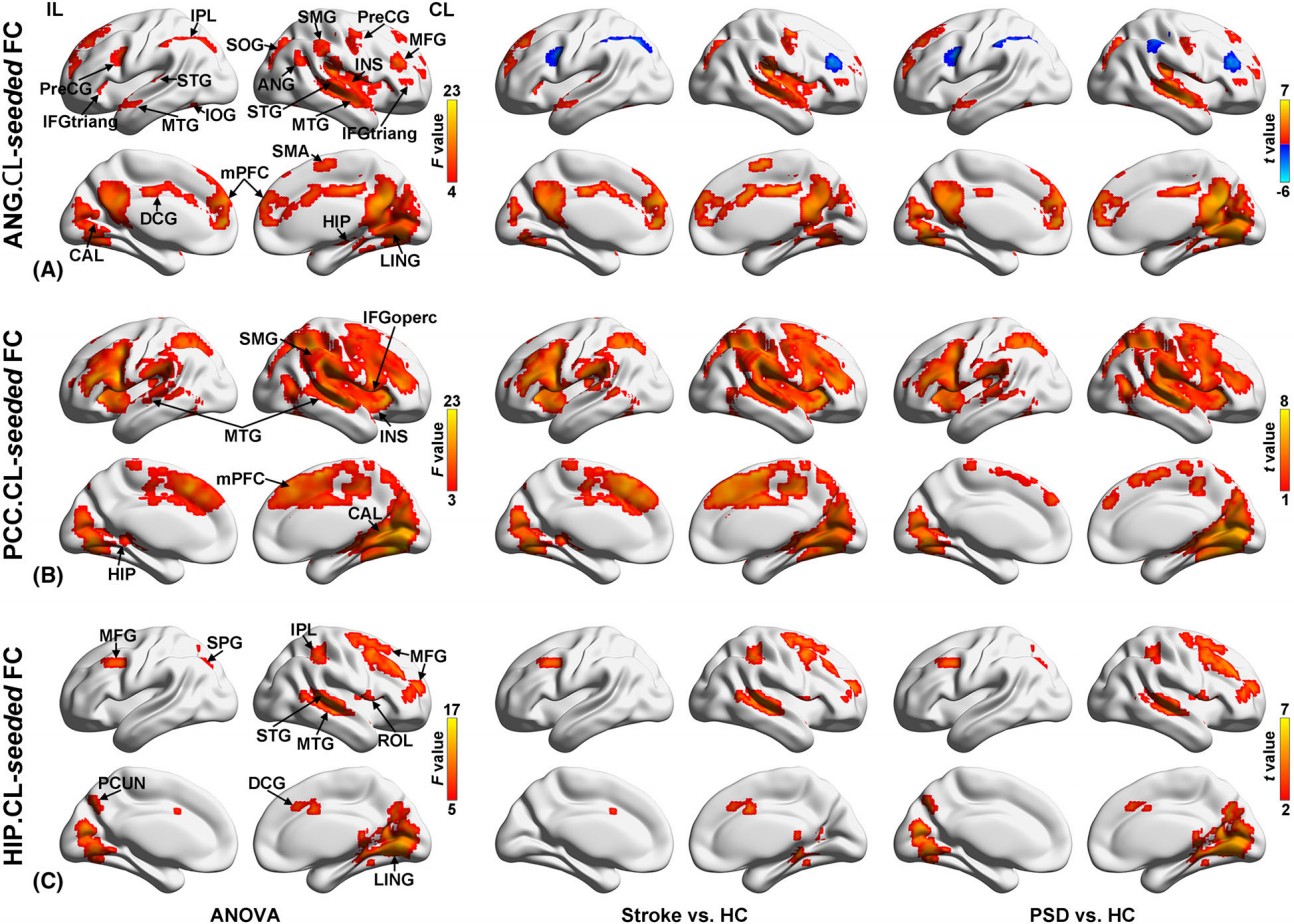
Group differences of seed‐based FC. Brian regions showing significant differences in contralesional ANG‐seeded (A), contralesional PCC‐seeded (B), and contralesional HIP‐seeded (C) FC among the three groups (left), between Stroke and HC groups (middle), and between PSD and HC groups (right) (*q* < 0.05, FDR corrected, k > 20 voxels). ANG, angular gyrus; CAL, calcarine; CL, contralesional hemishpere; DCG, median cingulate and paracingulate gyri; FC, functional connectivity; HC, healthy controls; HIP, hippocampus; IFGoperc, the opercular part of inferior frontal gyrus; IFGtriang, the triangular part of inferior frontal gyrus; IL, ipsilesional hemishpere; INS, insula; IOG, inferior occipital gyrus; IPL, inferior parietal; LING, lingual gyrus; MFG, middle frontal gyrus; mPFC, medial prefrontal cortex; MTG, middle temporal gyrus; PCC, posterior cingulate cortex; PCUN, precuneus; PreCG, precentral gyrus; PSD, poststroke depression; ROL, rolandic operculum; SMA, supplementary motor area; SMG, supramarginal gyrus; SOG, superior occipital gyrus; SPG, superior parietal gyrus; STG, superior temporal gyrus; Stroke, nondepressed stroke patients.

Using the contralesional ANG as the seed, the results of one‐way ANOVA showed that the brain regions exhibited significant among‐group differences in connectivity with contralesional ANG, involving bilateral STG, middle temporal gyrus (MTG), the medial prefrontal cortex (mPFC), the triangular part of inferior frontal gyrus (IFGtriang) and precentral gyrus (PreCG), ipsilesional inferior parietal (IPL), inferior occipital gyrus (IOG), calcarine (CAL), and median cingulate and paracingulate gyri (DCG), contralesional HIP, MFG, ANG, SMG, supplementary motor area (SMA), insula (INS), lingual gyrus (LING), and superior occipital gyrus (SOG) (*q* < 0.05, FDR corrected, k > 20 voxels) (Table S3 and Fig. 4A).

**Figure 5:**
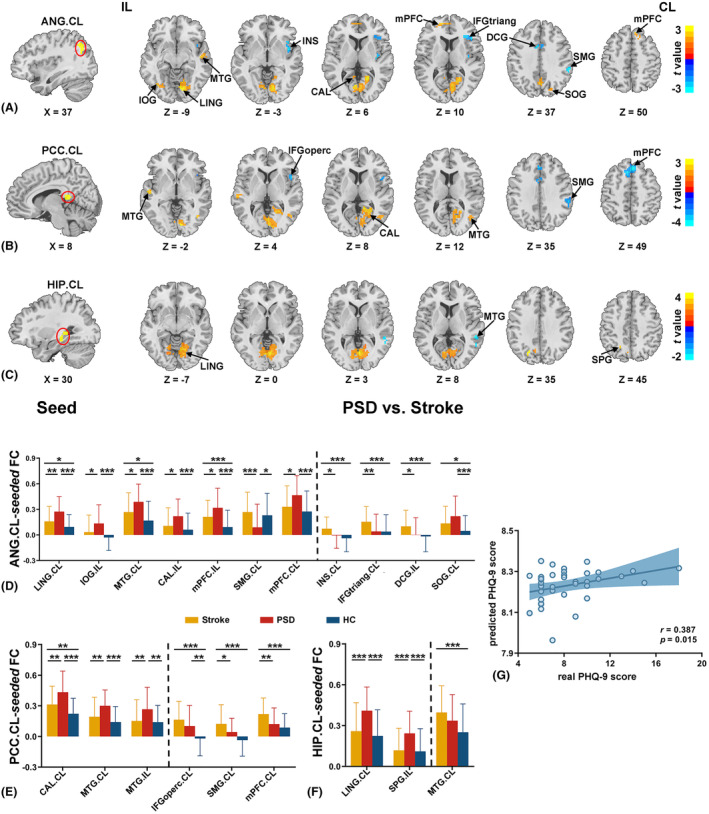
PSD‐specific region identified using seed‐based FC. The seed regions (left) and corresponding seed‐based FC resultant regions (right) where showed significant differences in contralesional ANG‐seeded (A), contralesional PCC‐seeded (B), and contralesional HIP‐seeded (C) FC between PSD and Stroke groups (*p* < 0.05, uncorrected, k > 20 voxels). Warm color indicates significantly higher FC value in PSD group than Stroke group. Cool color indicates significantly lower FC value in PSD group than Stroke group. The bar graph shows comparisons of average contralesional ANG‐seeded (D), contralesional PCC‐seeded (E), and contralesional HIP‐seeded (F) FC values between each pair of three groups in brain regions with significant differences between PSD and Stroke groups (*p* < 0.05, Bonferroni corrected). The regions on the left of dashed line showing PSD‐specific FC alterations. (G) Significant correlation was observed between real PHQ‐9 scores and the predicted PHQ‐9 scores. The predicted PHQ‐9 scores were obtained by the ridge regression model which using the average FC values of each region with PSD‐specific FC alterations in PSD group as the features (*p* < 0.017, Bonferroni corrected). *p < 0.05; **p < 0.01; ***p < 0.001. CAL, calcarine; CL, contralesional hemishpere; DCG, median cingulate and paracingulate gyri; FC, functional connectivity; HC, healthy controls; IFGoperc, the opercular part of inferior frontal gyrus; IFGtriang, the triangular part of inferior frontal gyrus; IL, ipsilesional hemishpere; INS, insula; IOG, inferior occipital gyrus; LING, lingual gyrus; mPFC, the medial prefrontal cortex; MTG, middle temporal gyrus; PHQ‐9, Patient Health Questionnaire‐9; PSD, poststroke depression; SMG, supramarginal gyrus; SOG, superior occipital gyrus; SPG, superior parietal gyrus; Stroke, nondepressed stroke.

The results of simple effect analyses revealed that the distributions with significant among‐group differences were mainly attributed to the differences with HC group. Compared with HC group, Stroke and PSD group showed analogous distribution of FC alterations, including increased connectivity with contralesional ANG in bilateral MTG, ipsilesional mPFC and IOG, contralesional STG, HIP, IFGtriang, PreCG.R, LING, and SOG, and decreased connectivity with contralesional ANG in ipsilesional PreCG and IPL, contralesional MFG (*q* < 0.05, FDR corrected, k > 20 voxels) (Fig. 4A). Compared with Stroke group, PSD group exhibited increased connectivity with contralesional ANG in bilateral mPFC, ipsilesional IOG and CAL, contralesional LING, MTG, and SOG, while decreased connectivity with contralesional ANG in ipsilesional DCG, contralesional INS, IFGtriang and SMG (*p* < 0.05, uncorrected, k > 20 voxels) (Table 3 and Fig. 5A). The subsequent comparisons between each pair of three groups in these regions observed PSD‐specific FC alterations in bilateral mPFC, ipsilesional IOG and CAL, contralesional LING, and MTG where PSD group exhibited increased FC, as well as in contralesional SMG, where PSD group exhibited decreased connectivity compared with both Stroke and HC groups (*p* < 0.05, Bonferroni corrected) (Fig. 5D).

**Table 3:** Brain regions showing significant differences in FC between PSD and Stroke groups.

Seed	Cluster	MNI coordinate (mm)	Cluster size	*t* value	Brain region
ANG.CL	1	12 −75 −9	515	3.8467	LING.CL
	2	−36 −69 −9	22	2.7569	IOG.IL
	3	48 −12 −12	32	2.9342	MTG.CL
	4	42 3 −3	36	−3.2807	INS.CL
	5	−9 −57 3	25	3.0663	CAL.IL
	6	45 24 9	31	−3.0692	IFGtriang.CL
	7	−18 54 9	39	2.7244	mPFC.IL
	8	−6 9 36	59	−3.2824	DCG.IL
	9	21 −75 42	24	2.677	SOG.CL
	10	57 −39 39	33	−3.6734	SMG.CL
	11	12 36 48	23	3.1955	mPFC.CL
PCC.CL	1	21 −54 6	297	3.6579	CAL.CL
	2	42 −66 −3	49	2.8826	MTG.CL
	3	−54 −15 −3	31	2.9003	MTG.IL
	4	48 15 3	23	−2.9596	IFGoperc.CL
	5	60 −39 30	67	−3.2465	SMG.CL
	6	6 30 45	220	−4.0291	mPFC.CL
HIP.CL	1	3 −69 3	512	3.7262	LING.CL
	2	54 −39 6	25	−2.4989	MTG.CL
	3	−24 −66 39	44	4.2404	SPG.IL

ANG, angular gyrus; CAL, calcarine; CL, contralesional hemisphere; DCG, median cingulate and paracingulate gyri; FC, functional connectivity; HIP, hippocampus; IFGoperc, the opercular part of inferior frontal gyrus; IFGtriang, the triangular part of inferior frontal gyrus; IL, ipsilesional hemisphere; INS, insula; IOG, inferior occipital gyrus; LING, lingual gyrus; MNI, Montreal Neurological Institute; mPFC, the medial prefrontal cortex; MTG, middle temporal gyrus; PCC, posterior cingulate cortex; PSD, poststroke depression; SMG, supramarginal gyrus; SOG, superior occipital gyrus; SPG, superior parietal gyrus; Stroke, nondepressed stroke patients.

#### Contralesional PCC‐seeded FC

One‐way ANOVA showed significant among‐group differences in contralesional PCC‐seeded FC in bilateral MTG, ipsilesional HIP and contralesional INS, CAL, the opercular part of inferior frontal gyrus (IFGoperc), SMG, and mPFC (*q* < 0.05, FDR corrected, k > 20 voxels) (Table S3 and Fig. 4B).

Simple effect analyses indicated that the distributions with significant among‐group differences were mainly attributed to the differences in comparison to HC group. Compared to HC group, Stroke and PSD group showed increased connectivity with the contralesional PCC in contralesional INS, CAL, MTG, IFGoperc, SMG, and mPFC (*q* < 0.05, FDR corrected, k > 20 voxels) (Fig. 4B). Compared to Stroke group, PSD group exhibited increased connectivity with the contralesional PCC in bilateral MTG and contralesional CAL, while decreased connectivity with the contralesional PCC in contralesional IFGoperc, SMG, and mPFC (*p* < 0.05, uncorrected, k > 20 voxels) (Table 3 and Fig. 5B). The further comparisons between each pair of three groups in these regions revealed PSD‐specific FC alterations in bilateral MTG and contralesional CAL, in which PSD group showed increased connectivity with contralesional PCC compared to both Stroke and HC groups (*p* < 0.05, Bonferroni corrected) (Fig. 5E).

#### Contralesional HIP‐seeded FC

Using the contralesional HIP as seed, one‐way ANOVA revealed that the brain regions exhibited significant among‐group differences in connectivity with the bilateral MFG, ipsilesional PCUN and superior parietal gyrus (SPG), contralesional LING, rolandic operculum (ROL), STG, DCG, IPL and MTG (*q* < 0.05, FDR corrected, k > 20 voxels) (Table S3 and Fig. 4C).

The results of simple effect analyses further showed that significant among‐group differences in HIP‐seeded FC were mainly attributed to the differences between stroke patients and HC group. Relative to HC group, Stroke and PSD groups exhibited increased connectivity with contralesional HIP in bilateral MFG, contralesional LING, ROL, STG, DCG, IPL, and LING (*q* < 0.05, FDR corrected, k > 20 voxels) (Fig. 4C). PSD group showed increased connectivity with contralesional HIP in ipsilesional SPG and contralesional LING while decreased connectivity in contralesional MTG when compared to Stroke group (*p* < 0.05, uncorrected, k > 20 voxels) (Table 3 and Fig. 5C). The subsequent comparisons between each pair of three groups in these regions detected PSD‐specific alterations of contralesional HIP‐seeded FC in ipsilesional SPG and contralesional LING, in which PSD group showed increased connectivity with contralesional HIP compared to both Stroke and HC groups (*p* < 0.05, Bonferroni corrected) (Fig. 5F).

Moreover, no significant correlations with onset time were observed for most of FC metrics in regions showing PSD‐specific FC alterations (*p* > 0.05) (Table S4).

### Connectivity–depression relationships in PSD

DC values in contralesional ANG positively correlated with the PHQ‐9 scores in PSD group (*r* = 0.420, *p* = 0.008) (Fig. 3C). No significant correlations with depression severity were observed for any other connectivity metrics (*p* > 0.017, Bonferroni corrected) (Tables S5 and S6).

The ridge regression analyses revealed that the average connectivity values in regions showing PSD‐specific FC alterations could predict PHQ‐9 scores (*r* = 0.387, *p* = 0.015) in PSD group (Fig. 5G and Supplementary Materials).

## Discussion

The present study applied connectivity‐based analyses to explore the specific alterations of intrinsic functional brain networks in PSD patients. The results showed that PSD was associated with increased functional connectivity strength (FCS) in some areas within the DMN compared to both Stroke and HC groups, which might be attribute to the specific alterations of connectivity between within DMN and outside DMN regions in PSD. Moreover, these connectivity changes correlated with and could predict the depression severity in PSD patients. Overall, these findings provide evidence for the disruptions of functional brain networks in PSD, which may aid in early diagnosis and interventions of the disease.

DC evaluates the FCS of the whole‐brain network at the voxel level. In the present study, the contralesional angular gyrus, posterior cingulate cortex, and hippocampus were found to show increased FCS in PSD patients compared to both Stroke and HC groups. ANG, has been reported to be one of the major connecting hubs in human brain networks, participates in diverse functions including memory retrieval and future simulation and semantic processing.^48^, ^49^ The abnormality of ANG has been consistently reported in PSD patients. Balaev and colleagues found the white matter volume of left ANG was associated with depression severity in PSD patients before treatment.^15^ Other studies demonstrated abnormal connectivity with ANG in PSD.^18^, ^50^ In addition, we found the increased DC in the contralesional ANG was positively correlated with PHQ‐9 scores in PSD. That is, the severer the depression in patients, the higher the DC values in the contralesional ANG. Posterior cingulate cortex (PCC), a highly metabolically active brain region,^51^, ^52^ is routinely engaged in cognitive tasks including self‐reflection^53^, ^54^ and autobiographical memory retrieval.^55^, ^56^ Our finding of increased DC in contralesional PCC in PSD was in accordance with recent studies which have consistently reported the alterations of connectivity with PCC in depressed patients.^15^, ^57^, ^58^, ^59^, ^60^, ^61^ For example, Oestreich and colleagues found that the connectivity with PCC was related to depressive symptoms in stroke patients.^61^ All these findings highlight the vital role of PCC in the occurrence of depression. Hippocampus is thought to be critical for memory,^62^, ^63^ emotional regulation,^64^ and contextual reward behavior.^65^ Voxel‐based morphometry studies have observed decreased hippocampal volumes in depressed patients.^66^, ^67^ In addition, our prior work has demonstrated increased activity in bilateral hippocampus in PSD patients.^11^ Thus, we propose that these structural and functional alterations in hippocampus may lead to the increased DC in contralesional hippocampus in PSD patients.

Notably, the ANG, PCC, and hippocampus are core components of DMN, which is a key brain network involved in spontaneous self‐generated thought patterns involving focus on the self and other people, memory, reward‐based decision‐making, and affective processing.^68^, ^69^ Based on our findings, we speculated that passive self‐reflection, and the deficits in memory processing and emotional regulation may contribute to increased node density and information transmission of these three regions in PSD patients. Moreover, the functional alterations of these three regions have also been reported in patients with major depressive disorder (MDD),^22^, ^57^, ^58^, ^70^ suggesting PSD may share a similar neural underpinnings with MDD. Hence, it is necessary for future work to clarify the similarities and differences between depressed patients caused by brain lesions and those who do not.

Using resting‐state FC analyses, the current study further explored the specific connectivity changes that could lead to the increase of DC in three regions in PSD. Our results showed that PSD patients increased connectivity between these three seeds and regions within DMN: bilateral MPFC and middle temporal gyrus and ipsilesional superior parietal gyrus. MPFC was associated with self‐referential processing^71^, ^72^ and emotion processing.^73^, ^74^ Animal models indicated that unilateral ischemic lesions of the medial prefrontal cortex can induce a pronounced and persistent depression phenotype.^75^ Middle temporal gyrus is a key region for emotion regulation and social perception,^76^, ^77^ its structural and functional abnormalities have been demonstrated in depressed patients.^78^, ^79^, ^80^ Superior parietal gyrus, as a part of parietal cortex, has been argued to support memory retrieval.^81^, ^82^ Hyperactivations in parietal cortex during the retrieval task have been observed in individuals with psychotic major depression.^82^ Our results of these increased connectivity were in accordance with the previous studies which consistently reported the disruptions of DMN connectivity in PSD patients.^9^, ^15^, ^16^, ^17^, ^18^, ^61^, ^83^, ^84^ For example, Zhang and colleagues found increased FC between PCC and left inferior parietal gyrus within DMN in PSD patients.^18^ These connectivity alterations within DMN were associated with depression scores and sensitive to rTMS treatment for PSD patients.^9^ All these findings together suggested that the increased FC within DMN may be associated with impaired emotional‐related function in PSD patients.

Meanwhile, the current study observed the increased connectivity between the three seeds and regions outside DMN, including bilateral calcarine, ipsilesional inferior occipital gyrus, and contralesional lingual gyrus, may also contribute to the increasement of DC in three seed regions within DMN in PSD patients. Increasing evidence from task fMRI studies have indicated the lingual gyrus may be involved in emotional preprocessing.^85^, ^86^ Structural MRI study has also demonstrated that the gray matter volume of lingual gyrus was larger in treatment responsive patients with MDD than in those nonresponsive patients and could predict early antidepressant response.^87^ Furthermore, the increased connectivity between the right anterior hippocampus of DMN and lingual gyrus in depressed patients compared to HCs has been reported in a recent study.^88^ Thus, we speculate that the increased connectivity between regions within DMN and lingual gyrus may result in depressed symptoms in PSD patients. Inferior occipital gyrus has been proposed to participate in mediating and appraising visually relevant, complex emotional stimuli.^89^, ^90^ Calcarine is related to vison‐processing and attentional modulation.^91^ Although the increased connectivity between the DMN regions and these two regions was not directly identified in previous studies, the functional abnormalities of these two regions have been reported to be associated with depression.^92^, ^93^, ^94^ Moreover, Eyre and colleagues applied the independent component analysis and revealed that the increasement of connectivity between DMN and visual network, which included occipital cortex in MDD patients.^95^ Notably, We found connectivity between contralesional ANG and ipsilesional inferior occipital gyrus was positively correlated with onset time in PSD groups. We speculate that it may be due to longer stroke onset time and transneuronal retrograde degeneration in PSD group.^96^, ^97^ Future studies are required to recruit patients with no differences in time after stroke onset to validate the alterations of connectivity between the DMN regions and these two regions in PSD patients.

In addition, the current study found decreased connectivity between the contralesional ANG and the contralesional supramarginal gyrus outside of DMN in PSD patients. Supramarginal gyrus is a key region for emotional and social processing,^98^, ^99^, ^100^, ^101^ and part of the neuroanatomical circuitry for sentence comprehension.^102^ Previous studies have demonstrated the impairments of connectivity between supramarginal gyrus and DMN in depressed patients.^103^, ^104^ Combining these findings and our results, we speculate that the decreased FC between contralesional ANG and supramarginal gyrus may be related to the inappropriate processing of negative emotional stimuli in PSD patients.

It is worth noting that these connectivity changes within DMN and between regions within and outside DMN could predict depression severity in PSD. This result further suggests that the connectivity alterations may serve as an index to monitor the degree of depression in PSD patients.

## Limitations

Several limitations should be mentioned in this study. Firstly, the current study was a cross‐sectional design that investigated abnormal functional brain network connectivity in PSD by recruiting patients within 1 month after stroke onset. Longitudinal studies are required to further explore how the patterns of connectivity changes related to the trajectory after stroke onset. Secondly, the differences between Stroke and PSD groups did not pass multiple comparison correction. Future studies with a larger sample size of PSD patients are needed to validate these findings of the current study. Thirdly, there exists a significant difference in onset time between the PSD and Stroke groups. Although no significant correlations with onset time were observed for most of connectivity metrics in regions showing PSD‐specific alterations. Future studies are required to recruit patients with no differences in time after stroke onset to validate the findings of this study. Finally, the depression severity of PSD patients in this study was within the range of mild to moderately severe depression (PHQ‐9). Future studies in PSD patients with different depression severity should further validate the prediction results of this study.

## Conflict of Interest

The authors declare no conflicts of interest.

## Author Contributions

Study concept and design: Xiumei Wu, Kang Xu, Yulin Song, and Yating Lv. Data acquisition, analysis, and interpretation: all authors. Drafting of the manuscript: Xiumei Wu, Kang Xu, Yulin Song, and Yating Lv. Critical review of manuscript: all authors.

## Supporting information


Appendix S1.
