# Supplementary material for: Abnormal intrinsic functional hubs and connectivity in patients with post‐stroke depression
Source: Ann Clin Transl Neurol. 2024 May 22;11(7):1852–67. doi: 10.1002/acn3.52091 (PMC11251479; doi:10.1002/acn3.52091)
Supplement: Supplementary file 1 — Appendix S1. [file ACN3-11-1852-s001.docx]

**Supplementary Materials**

**MRI data acquisition**

Within one month after stroke onset, all participants were scanned with a 3-Tesla scanner (GE MR-750, Waukesha, WI) at Anshan Changda Hospital with the following MRI scanning protocol: rs-fMRI, structural MRI (sMRI) and diffusion-weighted imaging (DWI). rs-fMRI data were collected using an echo-planar imaging sequence with axial slice = 43, slice thickness/gap = 3.2/0 mm, echo time (TE) = 30 ms, repetition time (TR) = 2000 ms, field of view (FOV) = 220 × 220 mm^2^, matrix size = 64 × 64, voxel size = 3.4 mm × 3.4 mm × 3.2 mm, flip angle (FA) = 90°, volumes = 240, time = 8’, and parallel acceleration = 2; sMRI data were obtained using a 3D-MPRAGE sequence with sagittal slice = 176, slice thickness/gap = 1/0 mm, TE = 3.1 ms, TR = 8100 ms, FOV = 256 × 256 mm^2^, matrix size = 256 × 256, voxel size = 1 mm × 1 mm × 1 mm, FA = 8°, time = 5’05”, prepare time = 450 ms, bandwidth = 31.25 kHz and parallel acceleration = 2; DWI data were acquired with axial slice = 22, slice thickness/gap = 5/1 mm, TR = 4000 ms, FOV = 240 × 240 mm^2^, b = 1000, time = 1’. During the MRI scanning, all participants were requested to keep the eyes closed, hold still, not fall asleep and not think about anything in particularly.

**Supplementary Table 1.** Brain regions showing significant differences in DC among three groups.

| **Cluster** | **MNI coordinate(mm)** | **Cluster size** | **F value** | **Brain region** |
| --- | --- | --- | --- | --- |
| 1 | -33 -72 0 | 2133 | 18.6253 | MOG.IL, ANG.CL, PCC.CL, HIP.CL, CUN.CL |
| 2 | -48 -39 9 | 56 | 9.6014 | STG.IL |
| 3 | 60 -21 3 | 27 | 11.8081 | STG.CL |
| 4 | 9 -27 69 | 1665 | 16.4506 | PoCG.IL, PCUN.IL |
| 5 | 30 9 36 | 86 | 15.7245 | MFG.CL |
| 6 | 60 -33 33 | 53 | 12.306 | SMG.CL |

***Abbreviations:*** DC, degree centrality; MNI, Montreal Neurological Institute; IL, ipsilesional hemisphere; CL, contralesional hemisphere; MOG, middle occipital gyrus; ANG, angular gyrus; PCC, posterior cingulate cortex; HIP, hippocampus; CUN, cuneus; STG, superior temporal gyrus; PoCG, postcentral gyrus; PCUN, precuneus; MFG, middle frontal gyrus; SMG, supramarginal gyrus.

**Supplementary Table 2.** Correlation between the PSD-specific alterations of DC values and onset time.

| **PSD-specific altered region** | **Stroke group: Onset time** | | |  | **PSD group: Onset time** | | |
| --- | --- | --- | --- | --- | --- | --- | --- |
|  | ***r* value** |  | ***p* value** |  | ***r* value** |  | ***p* value** |
| ANG.CL | 0.113 |  | 0.313 |  | -0.092 |  | 0.577 |
| PCC.CL | -0.068 |  | 0.546 |  | 0.103 |  | 0.532 |
| HIP.CL | 0.075 |  | 0.503 |  | 0.164 |  | 0.320 |

***Abbreviations:*** DC, degree centrality; Stroke, non-depressed stroke patients; PSD, post-stroke depression; CL, contralesional hemisphere; ANG, angular gyrus; PCC, posterior cingulate cortex; HIP, hippocampus.

**Supplementary Table 3.** Brain regions showing significant differences in FC among three groups.

| **Seed** | **cluster** | **MNI coordinate(mm)** | **Cluster size** | **F value** | **Brain region** |
| --- | --- | --- | --- | --- | --- |
| **ANG.CL** | 1 | 45 3 -15 | 871 | 22.2752 | STG.CL, MTG.CL, INS.CL |
|  | 2 | -45 -3 -15 | 97 | 16.3123 | MTG.IL |
|  | 3 | -3 54 6 | 2871 | 20.3227 | mPFC.IL, LING.CL, IOG.IL, CAL.IL, DCG.IL, SOG.CL |
|  | 4 | 27 -12 -12 | 42 | 11.52 | HIP.CL |
|  | 5 | -39 24 6 | 22 | 9.1094 | IFGtriang.IL |
|  | 6 | 39 33 3 | 73 | 12.5093 | IFGtriang.CL |
|  | 7 | -51 -24 12 | 31 | 8.4291 | STG.IL |
|  | 8 | 45 33 24 | 36 | 11.9993 | MFG.CL |
|  | 9 | 51 -57 24 | 21 | 6.2049 | ANG.CL |
|  | 10 | -48 12 30 | 54 | 11.1599 | PreCG.IL |
|  | 11 | -30 -63 39 | 196 | 13.8523 | IPL.IL |
|  | 12 | 51 -33 45 | 60 | 9.7789 | SMG.CL |
|  | 13 | 42 -12 42 | 43 | 7.4224 | PreCG.CL |
|  | 14 | 12 36 48 | 28 | 8.8089 | mPFC.CL |
|  | 15 | 6 -3 63 | 24 | 7.1971 | SMA.CL |
| **PCC.CL** | 1 | 48 9 -3 | 9150 | 22.8361 | INS.CL, CAL.CL, MTG.CL, MTG.IL, IFGoperc.CL, SMG.CL, mPFC.CL |
|  | 2 | -33 -33 0 | 49 | 7.6014 | HIP.IL |
| **HIP.CL** | 1 | 6 -66 -6 | 1097 | 16.9999 | LING.CL |
|  | 2 | 54 3 6 | 101 | 10.582 | ROL.CL |
|  | 3 | 57 -30 3 | 197 | 12.2431 | STG.CL, MTG.CL |
|  | 4 | 36 48 12 | 96 | 11.6025 | MFG.CL |
|  | 5 | 39 9 51 | 229 | 10.8189 | MFG.CL |
|  | 6 | 6 9 36 | 41 | 8.0393 | DCG.CL |
|  | 7 | -39 15 39 | 50 | 8.3807 | MFG.IL |
|  | 8 | -9 -69 42 | 60 | 7.3853 | PCUN.IL, SPG.IL |
|  | 9 | 45 -42 51 | 92 | 12.1722 | IPL.CL |

***Abbreviations:*** FC, functional connectivity; MNI, Montreal Neurological Institute; IL, ipsilesional hemisphere; CL, contralesional hemisphere; ANG, angular gyrus; PCC, posterior cingulate cortex; HIP, hippocampus; STG, superior temporal gyrus; MTG, middle temporal gyrus; INS, insula; mPFC, the medial prefrontal cortex; LING, lingual gyrus; IOG, inferior occipital gyrus; CAL, calcarine; DCG, median cingulate and paracingulate gyri; SOG, superior occipital gyrus; HIP, hippocampus; IFGtriang, the triangular part of inferior frontal gyrus; MFG, middle frontal gyrus; ANG, angular gyrus; PreCG, precentral gyrus; IPL, inferior parietal; SMG, supramarginal gyrus; SMA, supplementary motor area; IFGoperc, the opercular part of inferior frontal gyrus; ROL, rolandic operculum; PCUN, precuneus; SPG, superior parietal gyrus.

**Supplementary Table 4.** Correlation between the PSD-specific alterations of FC values and onset time.

| **Metric** | **PSD-specific altered region** | **Stroke group: Onset time** | | |  | **PSD group: Onset time** | | |
| --- | --- | --- | --- | --- | --- | --- | --- | --- |
|  |  | ***r* value** |  | ***p* value** |  | ***r* value** |  | ***p* value** |
| **Contralesional ANG-seeded FC** | LING.CL | 0.114 |  | 0.310 |  | 0.182 |  | 0.268 |
|  | IOG.IL | -0.012 |  | 0.917 |  | 0.384^*^ |  | 0.016 |
|  | MTG.CL | 0.053 |  | 0.638 |  | 0.040 |  | 0.807 |
|  | CAL.IL | 0.128 |  | 0.251 |  | 0.105 |  | 0.526 |
|  | mPFC.IL | -0.167 |  | 0.133 |  | 0.193 |  | 0.240 |
|  | SMG.CL | 0.020 |  | 0.858 |  | 0.112 |  | 0.496 |
|  | mPFC.CL | -0.012 |  | 0.917 |  | -0.133 |  | 0.421 |
| **Contralesional PCC-seeded FC** | CAL.CL | -0.083 |  | 0.457 |  | 0.021 |  | 0.898 |
|  | MTG.CL | -0.114 |  | 0.309 |  | 0.167 |  | 0.310 |
|  | MTG.IL | -0.103 |  | 0.359 |  | 0.105 |  | 0.523 |
| **Contralesional HIP-seeded FC** | LING.CL | -0.045 |  | 0.690 |  | 0.170 |  | 0.301 |
|  | SPG.IL | 0.060 |  | 0.592 |  | -0.004 |  | 0.983 |

***Abbreviations:*** FC, functional connectivity; Stroke, non-depressed stroke patients; PSD, post-stroke depression; IL, ipsilesional hemisphere; CL, contralesional hemisphere; ANG, angular gyrus; PCC, posterior cingulate cortex; HIP, hippocampus; LING, lingual gyrus; IOG, inferior occipital gyrus; MTG, middle temporal gyrus; CAL, calcarine; mPFC, the medial prefrontal cortex; SMG, supramarginal gyrus; SPG, superior parietal gyrus.

**Supplementary Table 5.** Correlation between the PSD-specific alterations of DC values and depression scale scores in PSD group.

| **PSD-specific altered region** | **PHQ-9** | |  | **HAMD** | |  | **CES-D** | |
| --- | --- | --- | --- | --- | --- | --- | --- | --- |
|  | ***r* value** | ***p* value** |  | ***r* value** | ***p* value** |  | ***r* value** | ***p* value** |
| ANG.CL | 0.420 | 0.008^**^ |  | 0.111 | 0.501 |  | 0.231 | 0.158 |
| PCC.CL | -0.032 | 0.846 |  | -0.173 | 0.293 |  | -0.022 | 0.895 |
| HIP.CL | -0.274 | 0.092 |  | -0.142 | 0.390 |  | -0.087 | 0.597 |

***Abbreviations:*** DC, degree centrality; PSD, post-stroke depression; PHQ-9, Patient Health Questionnaire-9; HAMD, Hamilton Depression Rating Scale; CES-D, Center for Epidemiological Survey Depression Scale; CL, contralesional hemisphere; ANG, angular gyrus; PCC, posterior cingulate cortex; HIP, hippocampus.

**Supplementary Table 6.** Correlation between the PSD-specific alterations of FC values and depression scale scores in PSD group.

| **Seed** | **PSD-specific altered region** | **PHQ-9** | |  | **HAMD** | |  | **CES-D** | |
| --- | --- | --- | --- | --- | --- | --- | --- | --- | --- |
|  |  | ***r* value** | ***p* value** |  | ***r* value** | ***p* value** |  | ***r* value** | ***p* value** |
| **ANG.CL** | LING.CL | 0.262 | 0.107 |  | 0.029 | 0.862 |  | 0.175 | 0.285 |
|  | IOG.IL | 0.230 | 0.159 |  | -0.026 | 0.877 |  | 0.123 | 0.457 |
|  | MTG.CL | 0.061 | 0.712 |  | -0.022 | 0.895 |  | 0.015 | 0.928 |
|  | CAL.IL | 0.229 | 0.160 |  | 0.066 | 0.690 |  | 0.176 | 0.285 |
|  | mPFC.IL | 0.196 | 0.232 |  | 0.035 | 0.834 |  | 0.193 | 0.240 |
|  | SMG.CL | 0.191 | 0.244 |  | -0.071 | 0.667 |  | -0.013 | 0.937 |
|  | mPFC.CL | 0.058 | 0.724 |  | -0.140 | 0.396 |  | 0.010 | 0.952 |
| **PCC.CL** | CAL.CL | -0.011 | 0.946 |  | 0.099 | 0.547 |  | 0.161 | 0.329 |
|  | MTG.CL | -0.122 | 0.460 |  | 0.029 | 0.859 |  | 0.044 | 0.790 |
|  | MTG.IL | -0.110 | 0.506 |  | -0.152 | 0.355 |  | -0.043 | 0.795 |
| **HIP.CL** | LING.CL | -0.308 | 0.056 |  | 0.052 | 0.755 |  | 0.033 | 0.840 |
|  | SPG.IL | 0.011 | 0.949 |  | 0.196 | 0.232 |  | 0.235 | 0.150 |

***Abbreviations:*** FC, functional connectivity; PSD, post-stroke depression; PHQ-9, Patient Health Questionnaire-9; HAMD, Hamilton Depression Rating Scale; CES-D, Center for Epidemiological Survey Depression Scale; IL, ipsilesional hemisphere; CL, contralesional hemisphere; ANG, angular gyrus; PCC, posterior cingulate cortex; HIP, hippocampus; LING, lingual gyrus; IOG, inferior occipital gyrus; MTG, middle temporal gyrus; CAL, calcarine; mPFC, the medial prefrontal cortex; SMG, supramarginal gyrus; SPG, superior parietal gyrus.

**The ridge regression analysis results**

$\hat{y}=0.024{LING.CL}^{a}+0.027{IOG.IL}^{a}+0.008{MTG.CL}^{a}+0.016{CAL.IL}^{a}+0.017{mPFC}^{a}+0.01{SMG.CL}^{a}+0.002{mPFC.CL}^{a}-0.002{CAL.CL}^{b}-0.008{MTG.CL}^{b}+0.003{MTG.IL}^{b}-0.028{LING.CL}^{c}+0.004{SPG.IL}^{c}$.

The equation shows the weights for FC features in the ridge regression model.

***Abbreviations:*** FC, functional connectivity; IL, ipsilesional hemisphere; CL, contralesional hemisphere; LING, lingual gyrus; IOG, inferior occipital gyrus; MTG, middle temporal gyrus; CAL, calcarine; mPFC, the medial prefrontal cortex; SMG, supramarginal gyrus; SPG, superior parietal gyrus; ANG, angular gyrus; PCC, posterior cingulate cortex; HIP, hippocampus.

^a^Contralesional ANG-seeded FC.

^b^Contralesional PCC-seeded FC.

^c^Contralesional HIP-seeded FC.
